# Supplementary material for: Half-quantized layer hall effect as a probe of quantized axion field
Source: Nat Commun. 2026 Jan 7;17:1305. doi: 10.1038/s41467-025-68071-y (PMC12868628; doi:10.1038/s41467-025-68071-y)
Supplement: Supplementary file 1 — Supplementary Information [file 41467_2025_68071_MOESM1_ESM.pdf]

Supplementary Information

Half-Quantized Layer Hall Effect as a Probe of Quantized Axion Field

Jiayuan Hu[1†], Binbin Wang[1†], Humian Zhou[2†], Tongtong Jia[3], Zheng Sun[3], Chang Liu[3], Bo Zhang[3], Dong Qian[3], Tingxin Li[3], X. C. Xie[2,4,5], Yunchuan Kong[1], Chui-Zhen Chen[6,7*] and Di Xiao[1*]

[1]Huawei Technologies Co., Ltd., Shanghai 201206, China

[2]International Center for Quantum Materials, School of Physics, Peking University, Beijing 100871, China

[3]State Key Laboratory of Micro-nano Engineering Science, School of Physics and Astronomy, Shanghai Jiao Tong University, Shanghai 200240, China

[4]Interdisciplinary Center for Theoretical Physics and Information Sciences, Fudan University, Shanghai 200433, China

[5]Hefei National Laboratory, Hefei 230088, China

[6]School of Physical Science and Technology, Soochow University, Suzhou 215006, China

[7]Institute for Advanced Study, Soochow University, Suzhou 215006, China

[†]These authors contributed equally: Jiayuan Hu, Binbin Wang, Humian Zhou.

[*]Corresponding authors: czchen@suda.edu.cn, xiaodi12@hisilicon.com

Supplementary Notes:

i. Transport data of a CBV Device with higher Cr doping

ii. Numerical simulation of the dependence of the Hall conductance on the magnetic field at different bottom gate voltages

iii. The HQLHE in devices with different spacer layer thicknesses

iv. Additional data of HQLHE in devices from different growths

v. The HQLHE of CBV devices in the n-doped regime

vi. A calculated phase diagram of LHE in an ideal CBV dual gated device

vii. The HQLHE in CBC devices

viii. Temperature dependence of HQLHE in CBV devices

ix. Basic characterization of the MBE grown CBV sandwich heterostructures

x. Geometries of the Hall bar devices

xi. Symmetrization and anti-symmetrization of the transport data

xii. Quantization deviation analysis

xiii. Discussion on other AHC mechanisms

xiv. Comparative analysis vs. layer Hall effect and the parity anomaly state

xv. Fermi level tuning efficiency by the bottom gate voltage

xvi. Additional raw data of the CBV devices

xvii. Characteristics of all devices measured in this study

Supplementary References

i. Transport data of a CBV Device with higher Cr doping

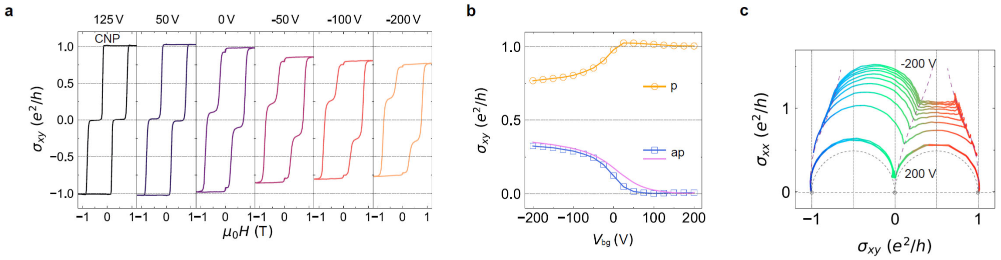

Supplementary Fig. 1 | Magnetoelectric transport data of a CBV Device A3 with higher Cr doping. (The same nominal parameters with Device A1, except for a much higher Cr doping level x = 0.36) **a,** Dependence of the Hall conductance σ_{xy} on the magnetic field $\mu_0 H$, measured at different bottom gate voltage V_{bg}. **b,** Dependence of the Hall conductance on V_{bg} with parallel (orange at zero field) and antiparallel (blue at ± 0.4 T; pink at zero field) magnetization configuration. **c,** Renormalization group flow in $(\sigma_{xy}, \sigma_{xx})$ plane with varying V_{bg} (-200 V to 200 V) and $\mu_0 H$ (± 1.2 T loop as in **a**). The purple dashed lines are guides to the eye for the flows from the fixed points $e^2/h\,(\pm 1.0, 0)$ and (0, 0) halfway towards points along $\sigma_{xy} = \pm e^2/2h$.

ii. Numerical simulation of the dependence of the Hall conductance on the magnetic field at different bottom gate voltages

In the CBV device A1, where both the top and bottom surfaces exhibit well-defined ferromagnetic order, the magnetization switching behavior of each surface (M_i, where $i = \mathrm{t}, \mathrm{b}$) is described by:

$$M_i^{<} = m_i \tanh\left(\frac{H + H_{C,i}}{H_{0,i}}\right) \text{ and } M_i^{>} = m_i \tanh\left(\frac{H - H_{C,i}}{H_{0,i}}\right) \qquad (1)$$

where "<" and ">" denote the positive-to-negative and negative-to-positive field sweeps, respectively. $\mu_0 H_{C,\mathrm{t}} = 0.12\ \mathrm{T}$ and $\mu_0 H_{C,\mathrm{b}} = 0.76\ \mathrm{T}$ are the coercive fields of the top (V-doped) and bottom (Cr-doped) surfaces. $\mu_0 H_{0,\mathrm{t}} = \mu_0 H_{0,\mathrm{b}} = 0.05\mathrm{T}$ characterizes the field scale for magnetization saturation. $m_{\mathrm{t}} = 0.3$ and $m_{\mathrm{b}} = 0.6$ represent the saturation magnetizations of the top and bottom surfaces, respectively.

In the VBC device C1, reduced V-doping level leads to a mixed state of weak ferromagnetism and superparamagnetism. Therefore, the magnetization of the bottom surface (the V-doped surface) is modeled by:

$$M_{\mathrm{b}}^{<} = m_{\mathrm{b}} \left[\mathrm{ptanh}\left(\frac{H + H_{C,\mathrm{b}}}{H_{0,\mathrm{b}}} \right) + (1-p) \tanh\left(\frac{H}{H_{1,\mathrm{b}}} \right) \right] \tag{2}$$

and

$$M_{\mathrm{b}}^{>} = m_{\mathrm{b}} \left[\mathrm{ptanh}\left(\frac{H - H_{C,\mathrm{b}}}{H_{0,\mathrm{b}}} \right) + (1-p) \tanh\left(\frac{H}{H_{1,\mathrm{b}}} \right) \right], \tag{3}$$

where p represents the fraction of ferromagnetic contribution. Unlike the bottom surface, the top surface (the Cr-doped surface) exhibits purely ferromagnetic behavior, hence the magnetization is modeled by:

$$M_{\mathrm{t}}^{<} = m_{\mathrm{t}} \tanh\left(\frac{H + H_{C,\mathrm{t}}}{H_{0,\mathrm{t}}} \right) \text{ and } M_{\mathrm{t}}^{>} = m_{\mathrm{t}} \tanh\left(\frac{H - H_{C,\mathrm{t}}}{H_{0,\mathrm{t}}} \right), \tag{4}$$

where m_{t} is the saturation magnetization, $H_{C,\mathrm{t}}$ is the coercive field, and $H_{0,\mathrm{t}}$ characterizes the field scale for the Cr-doped surface. In our numerical simulations of the magnetic field dependence of the Hall conductance in the VBC device, we adopt the following parameters based on experimental observations: $\mu_0 H_{C,\mathrm{t}} = 0.12$ T, $\mu_0 H_{C,\mathrm{b}} = 0.45$ T, $\mu_0 H_{0,\mathrm{t}} = 0.1$ T, $\mu_0 H_{0,\mathrm{b}} = 0.3$ T, $\mu_0 H_{1,\mathrm{b}} = 0.1$ T, $m_{\mathrm{t}} = 0.3$, $m_{\mathrm{b}} = 0.3$, and $p = 0.4$. Note that these parameters are used as an example to qualitatively demonstrate the dependence of σ_{xy} on the magnetic field.

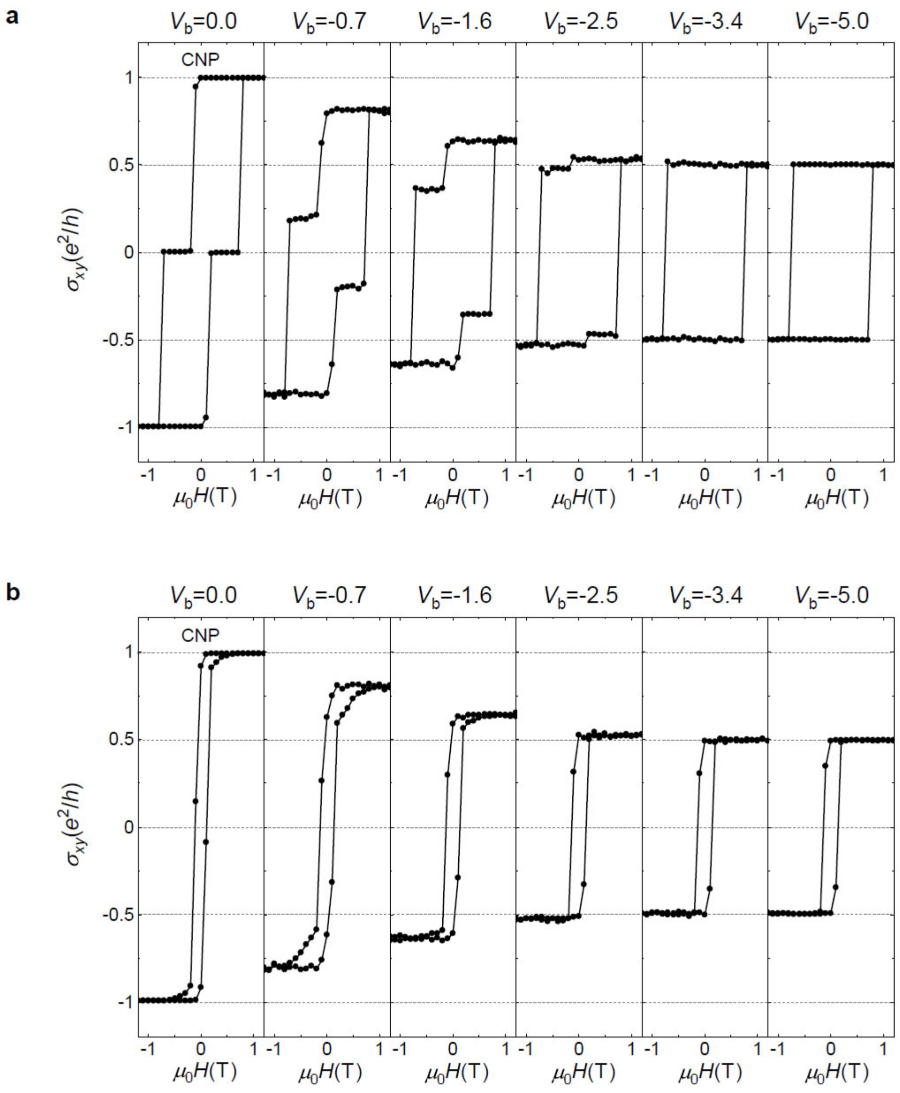

Supplementary Fig. 2 | Numerical simulation of the dependence of the Hall conductance σ_{xy} on the magnetic field at different V$_b$. a, Simulation for a CBV device with well-defined ferromagnetic order for both surfaces. **b,** Simulation for a VBC device with dominating superparamagnetism and weak ferromagnetism at the bottom V-doped surface. Both figures qualitatively fit well with the data in the main figures.

iii. The HQLHE in devices with different spacer layer thicknesses

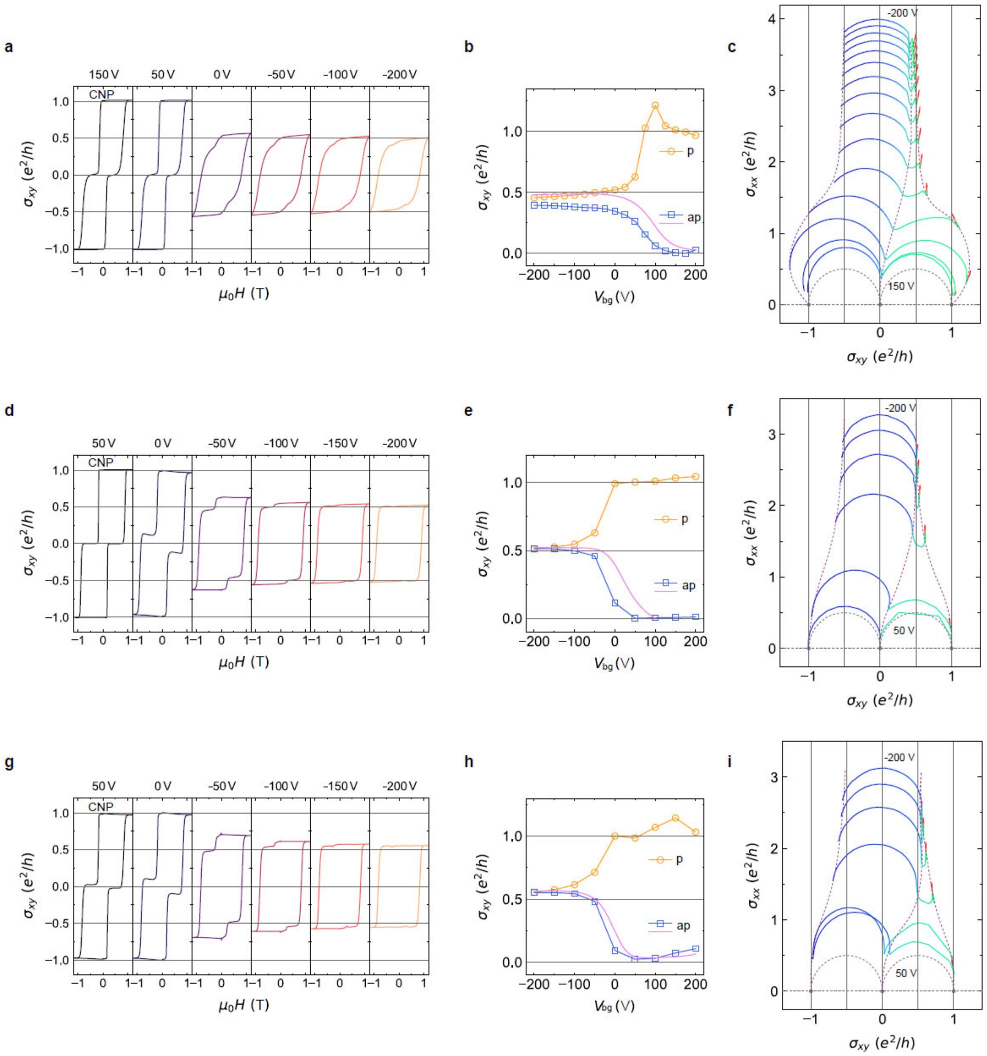

Supplementary Fig. 3 | The HQLHE in devices with different spacer layer thicknesses. a, d, g, Dependence of the Hall conductance σ_{xy} on the magnetic field $\mu_0 H$, measured at different bottom gate voltage V_{bg} on 3 CBV devices with different spacer layer thicknesses (**a**) Device A4, m = 5, (**d**) Device A5, m = 10, and (**g**) Device A6, m = 30. **b, e, h,** Dependence of the Hall conductance with parallel (orange circles, at zero field) and antiparallel (blue squares, at ± 0.4 T; pink line, at zero field) magnetization configuration on the bottom gate voltage V_{bg} for devices with (**b**) m = 5, (**e**) m = 10, and (**h**) m = 30. **c, f, i,** Renormalization group flow in $(\sigma_{xy}, \sigma_{xx})$ plane with varying V_{bg} (-200 V to CNPs) and $\mu_0 H$ (± 1.2 T loops as in **a, d, g**) for devices with (**c**) m = 5, (**f**) m = 10, and (**i**) m = 30. The m = 5 device has stronger hole doping

with effectively larger Cr concentration across the sandwich heterostructure, and stronger interlayer magnetic coupling due to its thin spacer. Thus, the AHC at $V_{\mathrm{bg}} = -200\ \mathrm{V}$ is slightly smaller than $e^2/2h$. The $m = 30$ device potentially has some quasi-1D nonchiral conduction channels on the side surfaces, which cause a slightly larger AHC at $V_{\mathrm{bg}} = -200\ \mathrm{V}$ and the conductance peak near $V_{\mathrm{bg}} = 150\ \mathrm{V}$. The $m = 10$ device shows better quantization in both QHLHE and QAHE regime, with $\sigma_{xy} = 0.51\ e^2/h$.

The anomalous Hall conductance is most accurate within a spacer thickness range of 10-20 QLs. This optimal range represents a critical trade-off between several competing physical effects.

For spacers thinner than $\sim$10 QLs, three possible issues arise. (**i**) The wavefunctions of the top and bottom surface states begin to overlap, leading to hybridization that opens a gap and destroys the layer-resolved half-quantized Hall conductance. This hybridization gap closes when the film thickness of Bi_2Te_3 type material is about ~5 QL[7]. The numerical results in Supplementary Fig. 4 support this analysis, showing that the layer-resolved Hall conductance is non-quantized for small thickness d due to hybridization effects, and gradually approaches half-quantization as d increases. (**ii**) Electrostatic screening is insufficient. The chemical potential of top (μ_{t}) and bottom (μ_{b}) surface satisfy $\mu_{\mathrm{t}}(V_{\mathrm{bg}}) = \mu_{\mathrm{b}}(V_{\mathrm{bg}})/\cosh(d/\lambda)$, where d is the thickness of the magnetic TI and λ is the Thomas–Fermi screening length of the bulk trap states. (See Supplementary Note xv for detailed derivation.) When $d < \lambda$, the back-gate voltage V_{bg} will shift the chemical potential of the top surface out of its local magnetic gap, leading to the breakdown of half-quantized layer Hall effect. (**iii**) The interlayer magnetic exchange coupling becomes significant, destabilizing antiparallel alignment of two magnetic layers and destroying the axion-insulator phase. All the above issues could be responsible for the observed reduced anomalous Hall conductance ~$0.48e^2/h$

and stronger interlayer magnetic coupling in Device A4 with spacer thickness of 5 QL (Supplementary Fig. 3a).

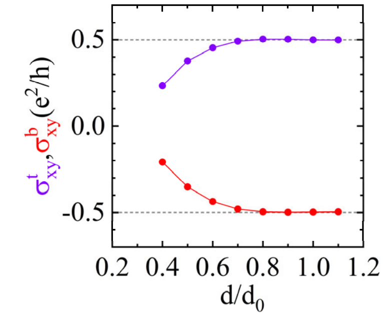

Supplementary Fig. 4 | Layer-resolved Hall conductance of axion insulators as a function of the TI thickness $\boldsymbol{d = N_z a_z}$ under the hybridization effect. In our calculations, we fix $N_z = 6$ and vary $\mathrm{a_z}$ to adjust d, where $d_0 = N_z a$.

For spacers thicker than ~20 QLs, the system begins to behave as two electronically decoupled films. In this limit, the quasi-1D metallic states on the side surface of TI, likely occur and contribute to the transport, leading to the deviations of half-quantized Hall conductance, see more details in Supplementary Note xii. Nonetheless, it is still possible to realize the HQLHE with spacers thicker than ~20 QLs, as long as the side surfaces maintain within the confinement gap.[8]

iv. Additional data of HQLHE in devices from different growths

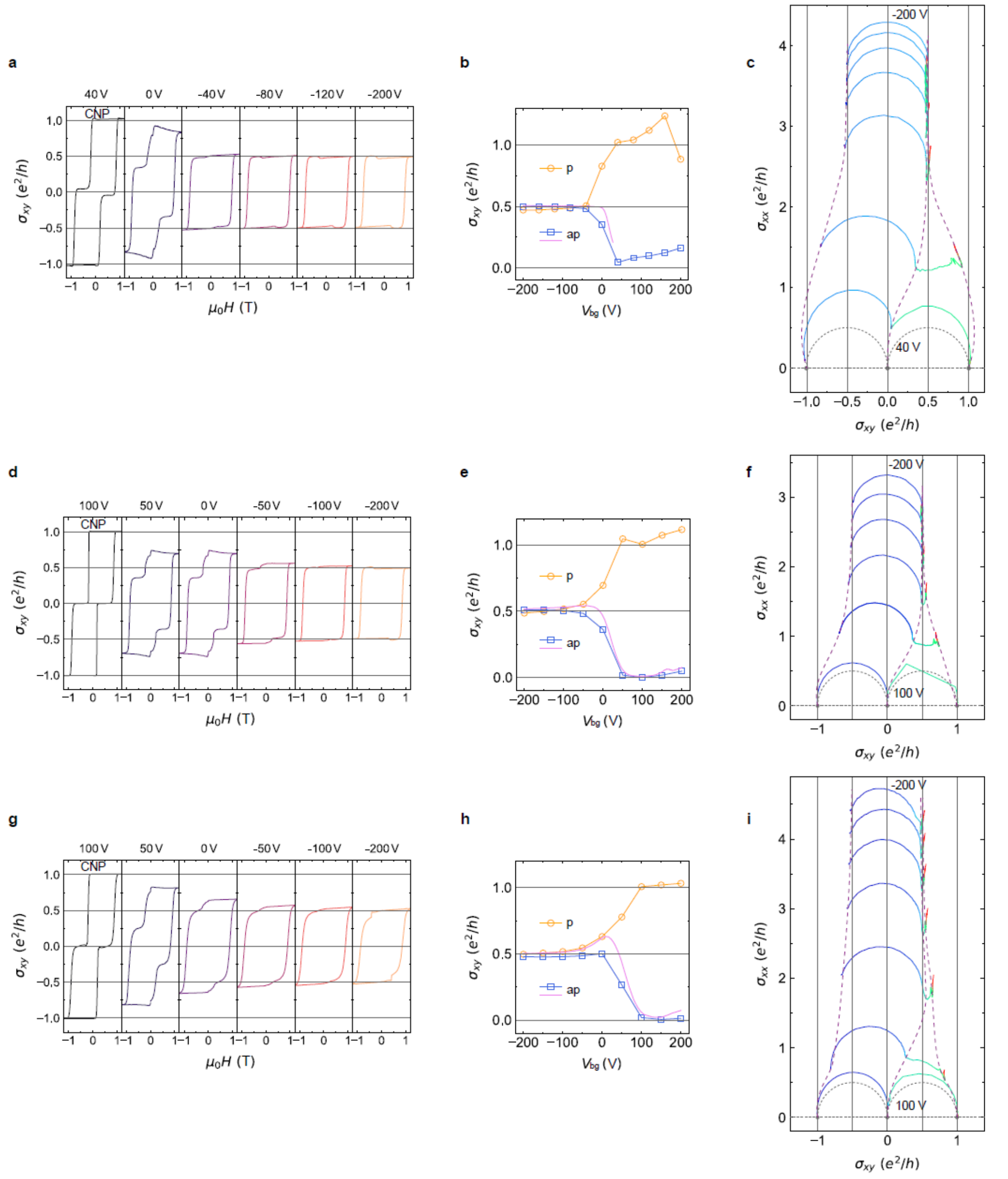

Supplementary Fig. 5 | Additional data of HQLHE in devices from different growths. The 3 CBV devices here have nominal parameters of (**a**)(**b**)(**c**) Device A2, m = 20, x = 0.19, y = 0.11, η = 0.83, $SrTiO_3$ thickness = 0.2 mm; (**d**)(**e**)(**f**) Device A7, m = 20, x = 0.19, y = 0.11, η = 0.83, $SrTiO_3$ thickness = 0.5 mm; (**g**)(**h**)(**i**) Device A8, m = 20, x = 0.19, y = 0.11, η = 0.62, $SrTiO_3$ thickness = 0.5 mm; **a, d, g,** Dependence of the Hall conductance σ_{xy} on the magnetic field $\mu_0 H$, measured at different bottom gate voltage V_{bg} on the 3 devices. **b, e, h,** Dependence of the Hall conductance with parallel (orange circles, at zero field) and antiparallel (blue squares, at $\pm$0.4 T; pink line, at

zero field) magnetization configuration on the bottom gate voltage V_{bg}. **c, f, i,** Renormalization group flow in $(\sigma_{xy}, \sigma_{xx})$ plane with varying V_{bg} (-200 V to CNPs) and $\mu_0 H$ (± 1.2 T loops as in **a, d, g**). All the devices show robust HQLHE with as V_{bg} approaching -200 V. The first device with reduced $SrTiO_3$ thickness of 0.2 mm seems to have a stronger modulation effect of Fermi level on the bottom surface, that the QHLHE's plateau (pink curve) is large with smallest deviation. The Hall conductance hysteresis of some devices (e.g. in **a** and **d** with $V_{\mathrm{bg}} = -200$ V) evolves further into another 2-step transition with inversed direction, because of the AHC sign reversal effect for magnetic TIs in the hole-doped regime. See Supplementary Note xiii.

v. The HQLHE of CBV devices in the n-doped regime

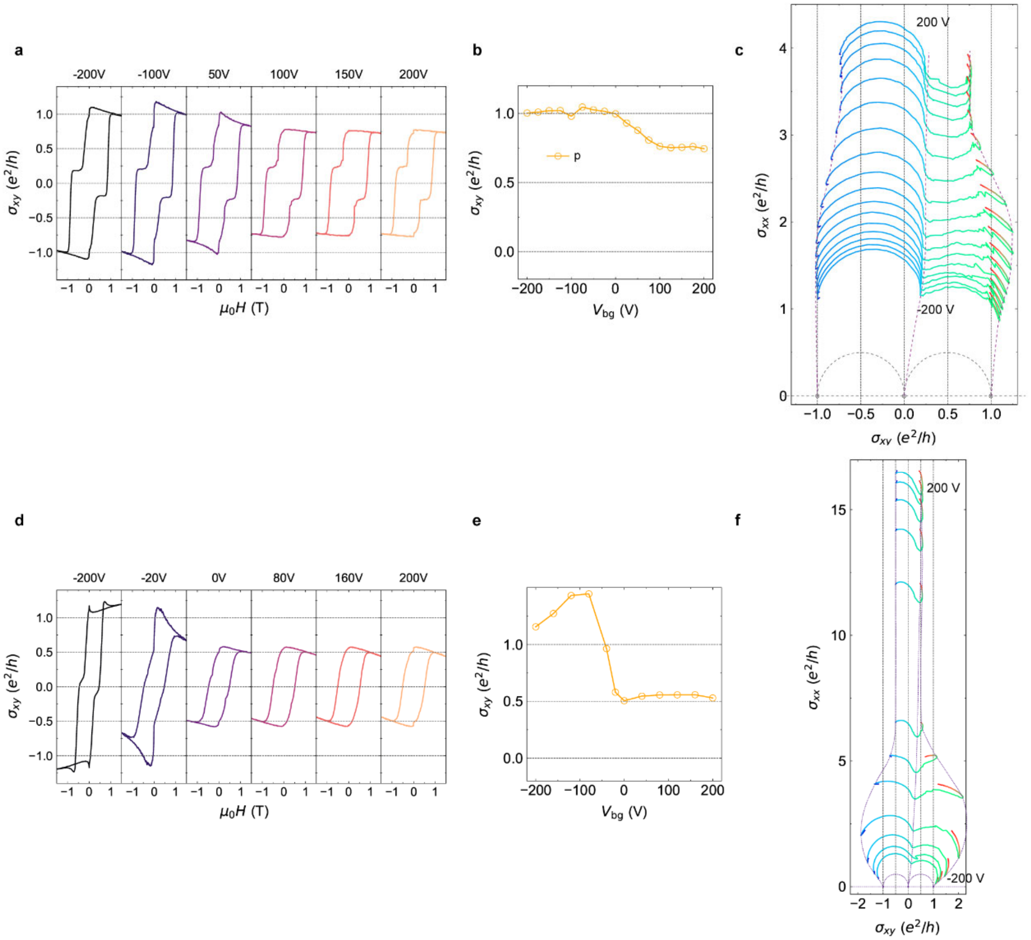

Supplementary Fig. 6 | The HQLHE of CBV devices in the n-doped regime. a, d, Dependence of the Hall conductance σ_{xy} on the magnetic field $\mu_0 H$, measured at different bottom gate voltage V_{bg} on 2 CBV devices with adjusted Bi:Sb ratio in the bottom part (3QL CBST - 10QL BST) of the heterostructure to approach a highly n-doped regime. (**a**) Device E1 with 3QL $Cr_{0.19}(Bi_{0.64}Sb_{0.36})_{1.81}Te_3$/10QL $(Bi_{0.64}Sb_{0.36})_2Te_3$/10QL $(Bi_{0.42}Sb_{0.58})_2Te_3$/3QL $V_{0.11}(Bi_{0.42}Sb_{0.58})_{1.89}Te_3$, (**d**) Device E2 with 3QL $Cr_{0.19}Bi_{1.81}Te_3$/10QL Bi_2Te_3/10QL $(Bi_{0.42}Sb_{0.58})_2Te_3$/3QL $V_{0.11}(Bi_{0.42}Sb_{0.58})_{1.89}Te_3$. **b, e,** Dependence of the Hall conductance with parallel (orange circles, at zero field) magnetization configuration on the bottom gate voltage V_{bg} for devices with (**b**) Device E1 and (**e**) Device E2. **c, f,** Renormalization group flow in $(\sigma_{xy}, \sigma_{xx})$ plane with varying V_{bg} (-200 V to 200 V) and $\mu_0 H$ (± 1.2 T loops as in **a, d**) for devices with (**c**) Device E1 and (**f**) Device E2. Although the n-doping is not strong enough for Device E1 to achieve the half-quantization, the AHC as a function of V_{bg} clearly demonstrate such a trend. Device E2 without Sb for the bottom part clearly shows a transition from $\sim e^2/h$ to a stable $\sim e^2/2h$ plateau, very similar to Fig. 2 in the original manuscript except for the opposite direction of tuning $V_{\mathrm{bg}} \to +200$ V. Because of the ferromagnetism now mediated by bulk carriers through RKKY mechanism, the coupling between the top and bottom surfaces becomes stronger so that a definitive antiparallel scenario is not well defined as shown in the hysteresis loops in **(d)**. We attribute the slight deviation from $e^2/2h$ near $V_{\mathrm{bg}} = 200$ V and the AHC peak near $V_{\mathrm{bg}} = -100$ V to the quasi-1D helical edge conduction channels as explained in Supplementary Note xii. The larger shift of chemical composition in Device E2 would possibly introduce some gap closing mechanism on the side surfaces, resulting in more conduction channels. Another possible source for the slightly larger AHC is the residual Berry curvature of CBT-BT layer due to the bulk conduction bands, as discussed in Supplementary Note xiii.

vi. A calculated phase diagram of LHE in an ideal CBV dual gated device

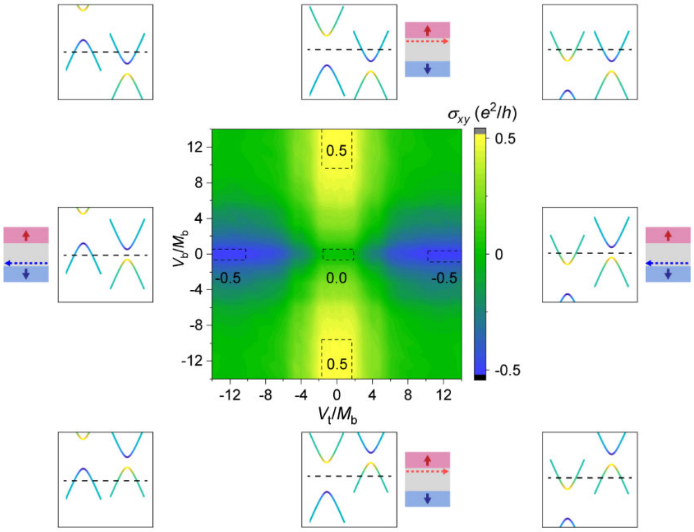

Supplementary Fig. 7 | A calculated phase diagram of LHE in an ideal CBV dual gated device with optimal gate efficiency. Calculated phase diagram for our axion insulator 4-band model, with σ_{xy} as a function of $V_{\mathrm{t}}/M_{\mathrm{b}}$ and $V_{\mathrm{b}}/M_{\mathrm{b}}$, assuming identical situations for both surfaces. The yellow ($\sigma_{xy} = e^2/2h$) and blue ($\sigma_{xy} = -e^2/2h$) regions indicated by the black dashed lines correspond to HQLHE originated from the top VBST layers and the bottom CBST layers, respectively, accompanying with schematics of the band alignment and Hall current distribution. The center green region defines the axion insulator regime with Fermi level locates inside the dual magnetic gaps. When the Berry curvature contributions from surfaces become relatively small, as indicated by the remaining greenish parts of the phase diagram, and 4 schematics on the corners, the AHC $\sigma_{xy} \approx 0$.

vii. The HQLHE in CBC devices

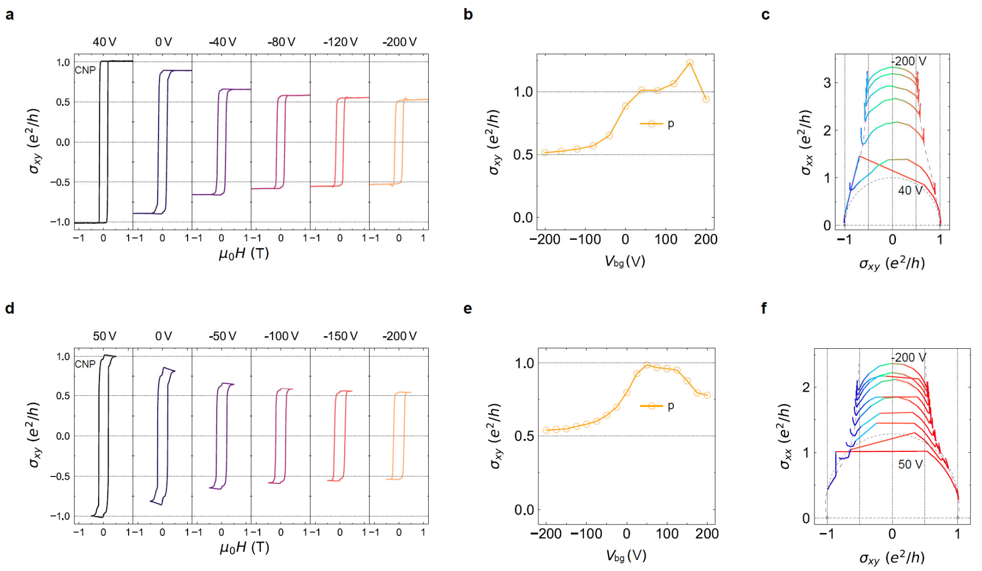

Supplementary Fig. 8 | The HQLHE in CBC devices. (**a**)(**b**)(**c**) Device D1 with m = 10, x = 0.19 for both top and bottom Cr doping, η = 0.83. (**d**)(**e**)(**f**) Device D2 with m = 20, x = 0.19, η = 0.83. **a,** Dependence of the Hall conductance σ_{xy} on the magnetic field $\mu_0 H$, measured at different bottom gate voltage V_{bg}. **b,** Dependence of the the Hall conductance on V_{bg} with parallel (orange at zero field) magnetization configuration. **c,** Renormalization group flow in $(\sigma_{xy}, \sigma_{xx})$ plane with varying V_{bg} (-200 V to 40 V) and $\mu_0 H$ ($\pm$1.2 T loops in **a**). **d,** Dependence of the Hall conductance σ_{xy} on the magnetic field $\mu_0 H$, measured at different bottom gate voltage V_{bg}. **e,** Dependence of the the Hall conductance on V_{bg} with parallel (orange at zero field) magnetization configuration. **f,** Renormalization group flow in $(\sigma_{xy}, \sigma_{xx})$ plane with varying V_{bg} (-200 V to 50 V) and $\mu_0 H$ ($\pm$0.5 T loops in **a**). The purple dashed lines in **c** and **f** are guides to the eye for the flows of the fixed points from $e^2/h\,(\pm 1.0, 0)$ towards points along $\sigma_{xy} = \pm e^2/2h$.

Notably, with symmetric magnetic doping, another trivial explanation for the half-quantization is that the total Hall conductance could accidentally reduce to $e^2/2h$ with contributions from both surfaces. This is unlikely because the bottom gate mainly

tunes the chemical potential of the bottom surface, as discussed in Supplementary Note xv. The smooth transition, as shown in the CBV-type devices upon applying V_{bg}, from a 2-step hysteresis loop (indicating contributions from both surfaces) to a single-loop hysteresis (contribution only from top surfaces) with a half-quantized AHC robustly excludes the trivial scenario.

viii. Temperature dependence of HQLHE in CBV devices

We assume that the Fermi level at the specific magnetic TI surface layers resides near the center of the exchange gap, which give rise to the half-quantized anomalous Hall conductance $\sigma_{xy} = e^2/2h$ at zero temperature. At finite temperatures, thermal excitation promotes charge carriers across the exchange gap, resulting in a measurable deviation of the Hall conductance from its quantized value. When thermal effects preserve both the exchange gap magnitude and Berry curvature topology, the change of temperature-dependent Hall conductance can be written as[13]:

$$\Delta\sigma_{xy} = -2\frac{e^2}{h}\int_{B.Z.}\frac{d^2k}{(2\pi)^2}\Omega(k)f(E), \tag{5}$$

where $\Omega(k)$ is the wavevector-dependent Berry curvature, $f(E)$ is the energy-dependent Fermi function. Combining with $\mathrm{E} = \sqrt{(\hbar v_f k)^2 + (\frac{\Delta}{2})^2}$ and $\Omega = \frac{\hbar^2 v_f^2 \Delta}{4E^3}$, the above equation can be simplified as

$$\Delta\sigma_{xy} = -\Delta\frac{e^2}{h}\int_{\frac{\Delta}{2}}^{\infty} dE\,\frac{f(E)}{E^2}, \tag{6}$$

where Δ is the heterostructure exchange gap. This equation enables quantitative determination of Δ through temperature-dependent Hall conductance measurements. As shown in Fig. 4 in the main text, the exchange gap Δ (0.77 meV) is extracted through the Hall conductance in the $0.1 - 3$ K regime, where we assume that the exchange gap is almost temperature-independent. At temperatures above 3 K, the AHC values drop more quickly due to thermally activated randomness of the dilute magnetic moments.

Notice that the assumption of the Fermi energy centered at the middle of the exchange

gap may not apply to all the devices in this study. In fact, devices with nominally the same doping concentration (usually indicating the same magnetic properties) often gives different Δ values. For example, the extracted Δ values of VBST layers of several different CBV devices with the same vanadium concentration y = 0.11, vary from 0.27~2.3 meV. We attribute these variations to the different Fermi level positions at the specific surfaces. When the Fermi level is near the gap edges, the extracted Δ value will be much smaller than the actual value. Therefore, the extracted Δ value by this method is only a lower bound on the actual exchange gap sizes. See Supplementary Fig. 9 for two more examples of temperature dependence of HQLHE.

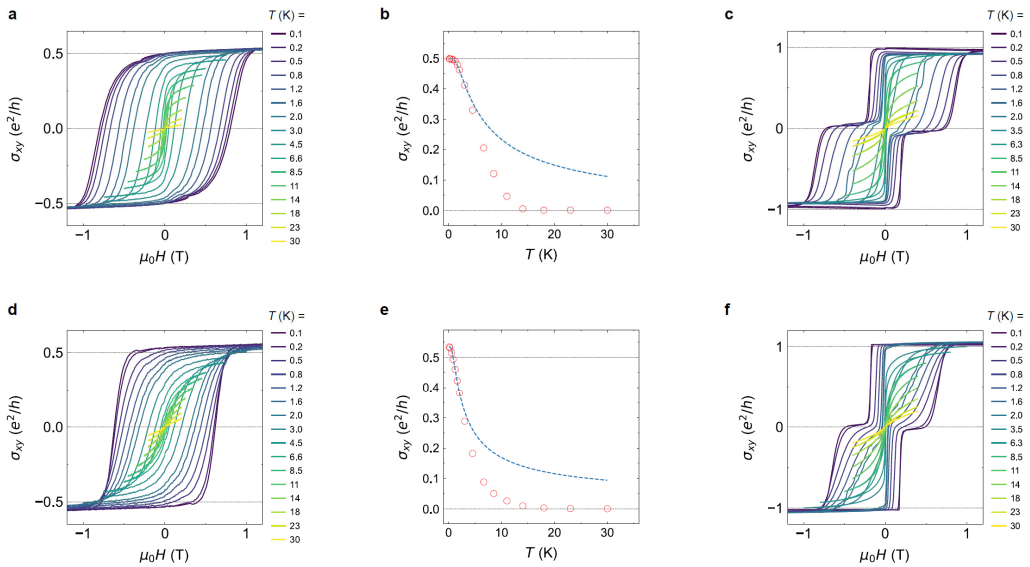

Supplementary Fig. 9 | Temperature dependence of HQLHE in CBV devices. (**a**)(**b**)(**c**) Device A5 with m = 10, x = 0.19, y = 0.11, η = 0.83. (**d**)(**e**)(**f**) Device A9 with m = 20, x = 0.19, y = 0.23, η = 0.83. **a,** Hall conductance loops of Device A5 as a function temperature at a fixed bottom gate voltage $V_{\mathrm{bg}} = -200\,V$. **b,** Temperature dependence of the AHC at zero magnetic field. The blue dashed line is the fit of the data at temperatures$\leq$ 3 K with the extracted exchange gap size Δ_{VBST}~0.66 meV. **c,** Hall conductance loops of Device A5 as a function temperature at CNP. **d,** Hall conductance loops of Device A9 as a function temperature at a fixed bottom gate

voltage $V_{\mathrm{bg}} = -200\ V$.**e,** Temperature dependence of the AHC at zero magnetic field. The blue dashed line is the fit of the data at temperatures$\leq$ 3 K with the extracted exchange gap size Δ_{VBST}~0.27 meV. **f,** Hall conductance loops of Device A9 as a function temperature at CNP.

ix. Basic characterization of the MBE grown CBV sandwich heterostructures

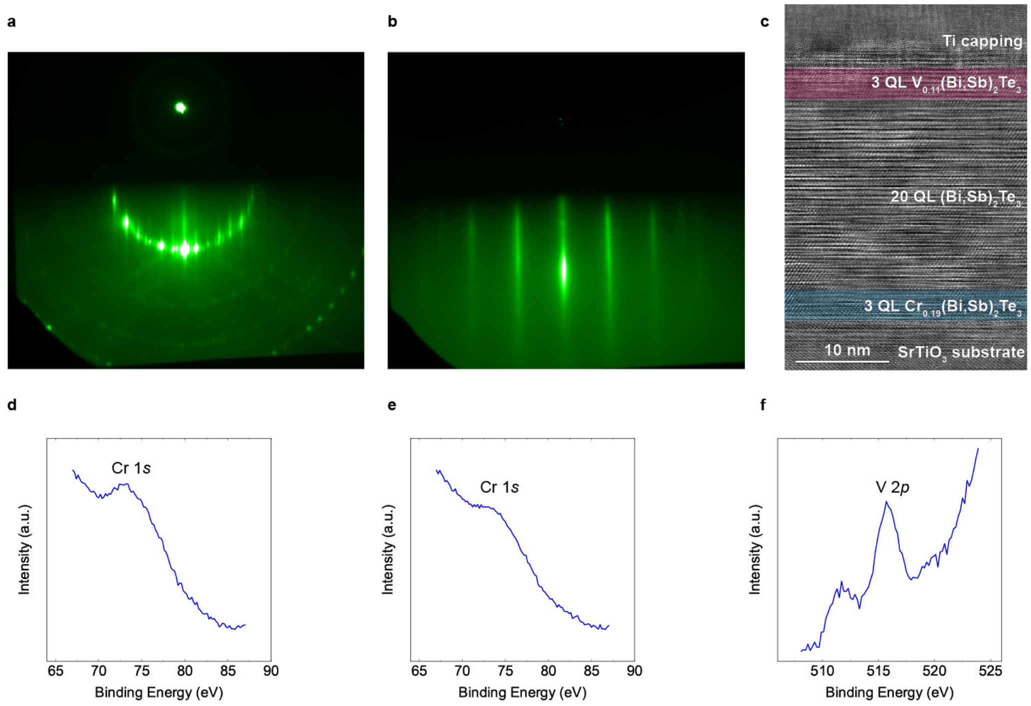

Supplementary Fig. 10 | Characterization of the MBE grown CBV sandwich heterostructures. a, RHEED pattern of a typical 2-step annealed SrTiO3 substrate prior to MBE growth of TI films. **b,** RHEED pattern of a CBV sample (m = 10, x = 0.19, y = 0.11, η = 0.83) after finishing the growth. The sharp and streaky 1x1 pattern is a sign of high-quality TI film. **c,** A transmission electron microscopy (TEM) image of a CBV sample (m = 20, x = 0.19, y = 0.11, η = 0.83). The signals from Cr (marked by blue region) and V (pink region) doping elements under the energy-dispersive spectroscopy are dim due to the dilute doping concentrations. The titanium capping on the surface caused intermixing of Ti and TI, obscuring the layered structure near the V-doped region. **d, e, f,** XPS data for the doping concentrations of calibration samples (with nominally the same growth parameters with Devices studied in the main text),

with fitting results of **d** $x = 0.19$, **e** $x = 0.34$, and **f** $y = 0.11$. The lower V concentration of Device C1 $y = 0.03$ is estimated according to the beam equivalent pressure (BEP) measurement instead.

x. Geometries of the Hall bar devices

As described in Methods, each scratched Hall bar dimensions (width W and length L) was measured using the optical microscope imaging system prior to low temperature transport measurements (a device is shown in Supplementary Fig. 11). We first conduct symmetrization and anti-symmetrization of the original data, as described in Supplementary Note xi, for $R_{xx} = V_{xx}/I_{ex}$, $R_{yx} = V_{yx}/I_{ex}$, respectively. Here, V_{xx} is the longitudinal voltage, V_{yx} is the Hall voltage, and I_{ex} is the excitation current (~10 nA). The (anti)symmetrized resistance data are then converted to resistivity using the relations $\rho_{xx} = \frac{W}{L} R_{xx}$ and $\rho_{yx} = R_{yx}$. Small measurement errors in the device geometries are inevitable. We estimate that the resulting uncertainty in the σ_{xy} (σ_{xy} is calculated using $\sigma_{xy} = \rho_{xy}/(\rho_{xx}^2 + \rho_{xy}^2)$) is less than 1.0%, suggesting that the observed deviations from half-quantization in the half-quantized layer Hall effect (HQLHE) state are likely due to other mechanisms as discussed in the main text and Supplementary Note xii.

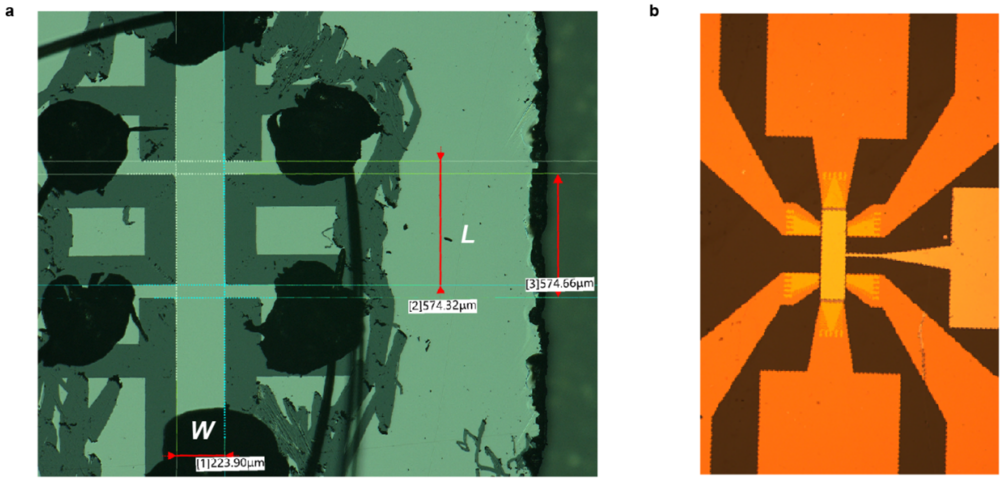

Supplementary Fig. 11 | Optical images of 2 type of Hall bar devices. a, Tungsten tip scratched Hall bar on a $SrTiO_3$ substrate. The dimensions were measured by the

optical imaging system, as indicated by the labels. The width W is 223.9 μm, and the length L is averaged 574.5 μm. **b,** Dual-gated Hall bar device fabricated through standard optical lithography, dry etching, and metal deposition processes.

xi. Symmetrization and anti-symmetrization of the transport data

We employ standard symmetrization and anti-symmetrization procedures on the magneto-transport data to eliminate the mixing of longitudinal and Hall voltage signals. Unlike in the QAH case where the bulk is strictly insulating, the HQLHE exhibits a finite longitudinal conductance (usually 2~4 h/e^2), which will largely change the measured value of Hall resistance due to imperfect device geometries. As described above, although the Hall bar devices are carefully etched or scratched, such mixing can still occur due to the slight misalignment. Note that when the device is at the CNP (axion insulator), even small variations in temperature (a few mK) during measurements may introduce instability in the resistance data (e.g. some curves in Supplementary Fig. 12a and 13a). However, these variations do not affect the calculated conductance results (both σ_{xx} and σ_{xy} almost vanish near CNP).

Therefore, the raw data are processed using the following procedures. For the magnetic field scan:

$$R_{xx}(\mu_0 H; \rightarrow) = \frac{1}{2}[R_{xx}(\mu_0 H; \rightarrow) + R_{xx}(-\mu_0 H; \leftarrow)]; \tag{7}$$

$$R_{yx}(\mu_0 H; \rightarrow) = \frac{1}{2}\left[R_{yx}(\mu_0 H; \rightarrow) - R_{yx}(-\mu_0 H; \leftarrow)\right]. \tag{8}$$

here, $\rightarrow$ and $\leftarrow$ denote the scanning direction of the magnetic field, and $\mu_0 H$ is the magnetic field. See Supplementary Fig. 12 for an example of symmetrization and anti-symmetrization of Device A1's (m = 20 QL, x = 0.19, y = 0.11, η = 0.83) data of magnetic field scan.

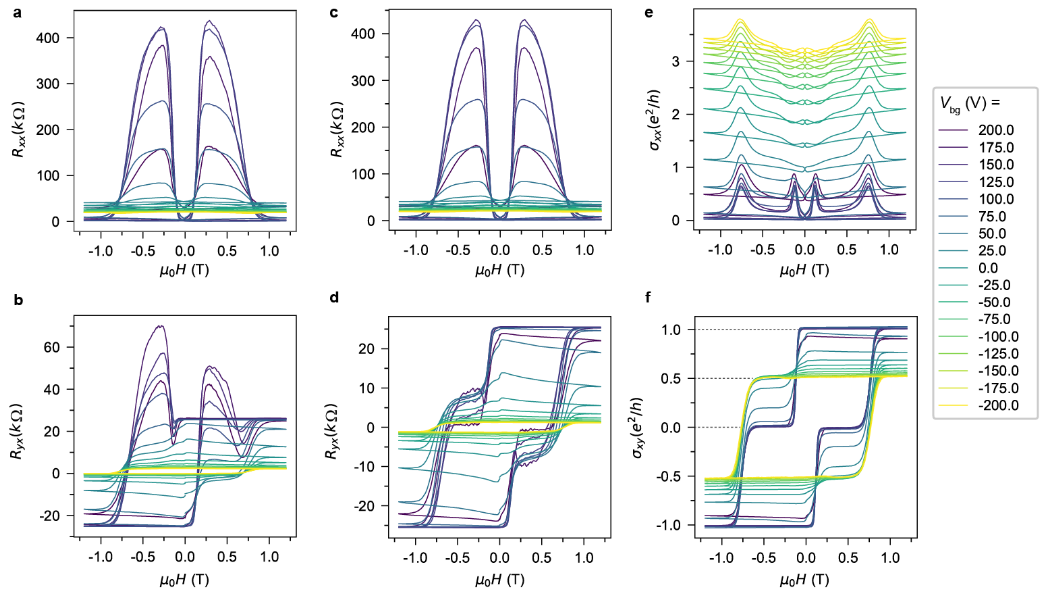

Supplementary Fig. 12 | Symmetrization and anti-symmetrization of the magnetic field scan data. a, b, The original R_{xx} and R_{yx} vs magnetic field data for Device A1, measured at various bottom gate voltage V_{bg}. **c, d,** R_{xx} and R_{yx} vs magnetic field, after symmetrization and anti-symmetrization, respectively. **e, f,** σ_{xx} and σ_{xy} vs magnetic field calculated from processed data in **c** and **d**.

For the bottom gate voltage scan at zero magnetic field, in addition to the mixing effect of longitudinal and Hall data, the bottom gate dielectric SrTiO3 itself has some hysteresis effect. We overcome this effect by training the bottom gate from 200 V to -200 V several times before taking the data. We also take at least two traces of data at the same scanning speed and direction in order to check if these two runs of data overlap well. Then we choose the last run as the final accepted raw data. For the final process of symmetrization/anti-symmetrization:

$$R_{xx}(V_{\mathrm{bg}};\uparrow) = \frac{1}{2}\left[R_{xx}(V_{\mathrm{bg}};\uparrow) + R_{xx}(V_{\mathrm{bg}};\downarrow)\right]; \quad (9)$$

$$R_{yx}(V_{\mathrm{bg}};\uparrow) = \frac{1}{2}\left[R_{yx}(V_{\mathrm{bg}};\uparrow) - R_{yx}(V_{\mathrm{bg}};\downarrow)\right]. \quad (10)$$

where $\uparrow$ and $\downarrow$ denotes one magnetization configuration and its time-reversal counterpart, and V_{bg} is the bottom gate voltage. See Supplementary Fig. 13 for an example of symmetrization and anti-symmetrization of Device A1's data of bottom gate

voltage scan. The magnetic field were trained from parallel (+1.2 T) to antiparallel (-0.4 T to 0; configuration ↑) to take data, then trained to parallel (-1.2 T) and finally to antiparallel (+0.4 T to 0; configuration ↓) to take the second half of data.

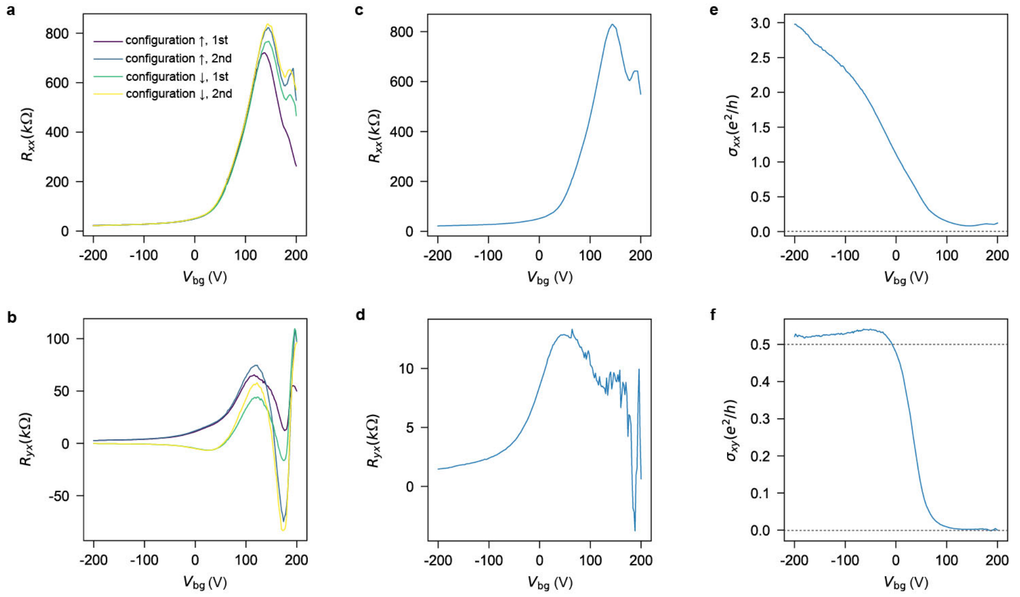

Supplementary Fig. 13 | Symmetrization and anti-symmetrization of the transport data. a, b, The original R_{xx} and R_{yx} vs bottom gate voltage V_{bg} data of Device A1. After gate training, 2 consecutive runs were taken from 200 V to -200V at each magnetic configuration. Here ↑(↓) denotes antiparallel with surface magnetization direction pointing outwards (inwards). **c, d,** R_{xx} and R_{yx} vs bottom gate voltage data after symmetrization and anti-symmetrization from the 2nd scan in **a** and **b** respectively. **e, f,** σ_{xx} and σ_{xy} vs bottom gate voltage calculated from processed data in **c** and **d**.

xii. Quantization deviation analysis

We attribute the quantization deviations in both HQLHE and QAHE to quasi-1D helical edge conduction channels. Additional conductance peaks near QAHE's $\sigma_{xy} = e^2/h$ plateau and AHC deviation with $|\sigma_{xy}| > e^2/2h$ often coexist in the CBV axion insulator devices. Supplementary Fig. 14 depicted a typical behavior described above. Intriguingly, there are conductance fluctuations that are most prominent when the bottom gate voltage locates near the AHC peaks ($V_{\mathrm{bg}} = -100\,\mathrm{V}$ and $40\,\mathrm{V}$ in

Supplementary Fig. 14g), and almost vanish near QAHE plateau ($V_{\mathrm{bg}} = -20$ V Supplementary Fig. 14h and i). This can be understood from the presence of quasi-1D helical edge conductions. Note that the magnetic doping on the top and bottom surfaces does not introduce any gap on the side surfaces. Therefore, these channels are more likely to form when the device's side surfaces become larger as the spacer thickness increases, due to weakened confinement effect. The presence of this additional transport features is occasional, which can be explained by the Fermi level's accidental crossing with the energy levels on the side surfaces. These conductance fluctuations gradually fade away at temperature above $\sim$1 K, which suggests that they are quantum effect potentially related to universal quantum fluctuations[1], instead of thermal noise. Detailed study of these conductance fluctuations is beyond the scope of this work and will be reported elsewhere.

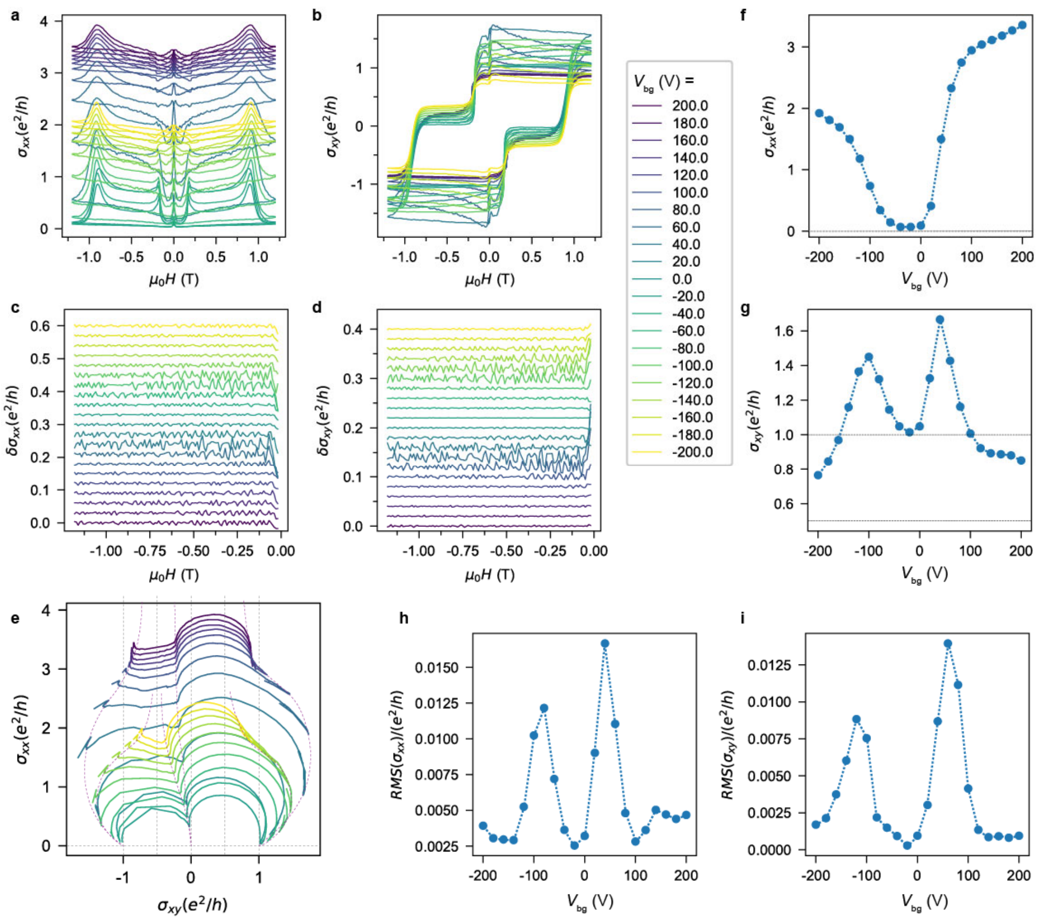

Supplementary Fig. 14 | Deviation from quantization, conductance peaks and quantum fluctuations caused by quasi-1D helical edge conduction. a, b, The longitudinal conductance σ_{xx} (**a**) and Hall conductance σ_{xy} (**b**) vs magnetic field data of a CBV Device A10 (x = 0.19, y = 0.11, m = 10, η = 0.73) and **c, d,** The conductance fluctuations extracted from data in **a** and **b** by subtracting a smoothed background. **e,** RGFD of Device A10 reveals the trend of the flow towards HQLHE on both electron and hole doped regimes as the bottom gate voltage tuning away from the CNP. The fixed point $e^2/h\,(-1.0, 0)$ and (0, 0) flow towards points along $\sigma_{xy} = -e^2/2h$, while the fixed point $e^2/h\,(+1.0, 0)$ flowing towards points along $\sigma_{xy} = +e^2/2h$. **f, g,** The longitudinal conductance σ_{xx} (**a**) and Hall conductance σ_{xy} (**b**) at zero magnetic field vs bottom gate voltage. The QAH with $\sigma_{xx} = 0$, $\sigma_{xy} = e^2/h$ is present at CNP with $V_{\mathrm{bg}} = -25$ V. There are additional conductance peaks on both sides of the C = 1 plateau. **h, i,** the root mean square (RMS) of the conductance fluctuations, exhibiting the most prominent fluctuations locates near the 2 additional conductance peaks.

To study the influence of helical edge states on the quantization deviation of the experimentally measured Hall conductance, we construct a conductor network model[2–5] for a system of size $L_x \times L_y$ with Hall conductivity σ_{xy} and longitudinal conductivity σ_{xx}. When the sample size exceeds the dephasing length l_ϕ, quantum coherence is lost at larger scales. Thus, the system can be treated as a network of independent classical resistive blocks, each of size L_0 (where $L_0 > l_\phi$). Since quantum interference effects are negligible beyond l_ϕ, these blocks behave as classical resistors connected via Ohm's law. Within the Landauer formalism, we model the network by introducing transmission coefficients $T_{p,q}$ between adjacent blocks p and q (see Supplementary Fig. 15a). For nearest-neighbor blocks in the bulk, the transmission is symmetric: $T_{p,q} = T_{q,p} = t_n$. However, at the edges, chirality and

helical modes introduce asymmetry: we have $T_{p+\hat{e}_x,p} = t_n + t_{1\mathrm{D}}$ and $T_{p,p+\hat{e}_x} = t_n + t_{1\mathrm{D}} + t_d$ in the upper edge, and $T_{p+\hat{e}_x,p} = t_n + t_{1\mathrm{D}} + t_d$ and $T_{p,p+\hat{e}_x} = t_n + t_{1\mathrm{D}}$ in the lower edge. Here, $p + \hat{e}_x$ denotes the block to the right of p. The transmission coefficients originate from three distinct conduction channels: $t_n = \frac{h}{e^2}\sigma_{xx}$ (bulk metallic channels), $t_d = \frac{h}{e^2}\sigma_{xy}$ (chiral edge channels), and $t_{1\mathrm{D}} = MT/(1-T)$ (helical edge channels). Here, M counts the number of helical edge modes, and T gives the electron transmission probability between nearest-neighbor blocks through these helical edge channels.

Given the transmission coefficients between blocks, the six-terminal measured Hall conductivity $\sigma_{xy}^{\mathrm{exp}}$ can be numerically calculated using the six-terminal Landauer-Büttiker formalism. According to Landauer-Büttiker formula[6], the current in the lead p can be expressed as:

$$I_p = \frac{e^2}{h}\sum_{q\neq p}\left(T_{qp}V_p - T_{pq}V_q\right), \tag{11}$$

where V_p is the voltage in the lead p. In our calculations, the real lead 1 and the real lead 4 act as current electrodes and other real leads act as voltage electrodes. Thus, the longitudinal current $I_1 = -I_4 \equiv I_x$ when an external bias is applied between lead 1 and lead 4 with $V_1 = V/2$ and $V_4 = -V/2$. The net current of the blocks is zero due to current conversation. Combining Landauer-Büttiker formula with those boundary conditions in the real and the blocks, one can calculate the voltage of each real lead and the longitudinal current I_x. Then the Hall resistance $\rho_{xy} = (V_2 - V_6)/I_x$ and longitudinal resistance $\rho_{xx} = (V_2 - V_3)/I_x(L_y/L)$ will be obtained. The six-terminal measured Hall conductivity is given by $\sigma_{xy}^{\mathrm{exp}} = \rho_{xy}/(\rho_{xx}^2 + \rho_{xy}^2)$.

As shown in Supplementary Fig. 15b, nonzero $t_{1\mathrm{D}}$ causes $\sigma_{xy}^{\mathrm{exp}}$ to exceed σ_{xy}, explaining the deviation from quantized Hall conductance in experimental measurements. $\sigma_{xy}^{\mathrm{exp}}/\sigma_{xy}$ first goes up and then drops as t_n increases. When $t_n \gg$

t_{1D}, the influence of t_{1D} on the transport is negligible so $\sigma_{xy}^{\exp}/\sigma_{xy} = 1$. In order to explain the peak of Hall conductance peaks near QAHE's in Supplementary Fig. 14g, we numerically calculate the intrinsic Hall conductance σ_{xy} of the magnetic TI by Kubo formula, and hence calculate the $\sigma_{xy}^{\exp}$ by Landauer-Büttiker formula (see Supplementary Fig. 15c). We use $t_n = 0.45|E_f/m_b|\Theta(|E_f| - m_1)$ with mobility gap $m_1 = 0.07$ to capture the main feature of the longitudinal conductance in experimental results, and $M = 4\Theta(|\mathrm{V_b}| - m_2)$ with fine size gap $m_2 = 0.08$. When $|V_\mathrm{b}| < m_2$, there is no helical edge states, so the measured Hall conductance $\sigma_{xy}^{\exp}$ is quantized. When $|V_\mathrm{b}| > m_2$, the helical edge states participate in transport and t_{1D} becomes nonzero. Therefore, we see $\sigma_{xy}^{\exp}$ first goes up and then drops as $|V_\mathrm{b}|$ increases.

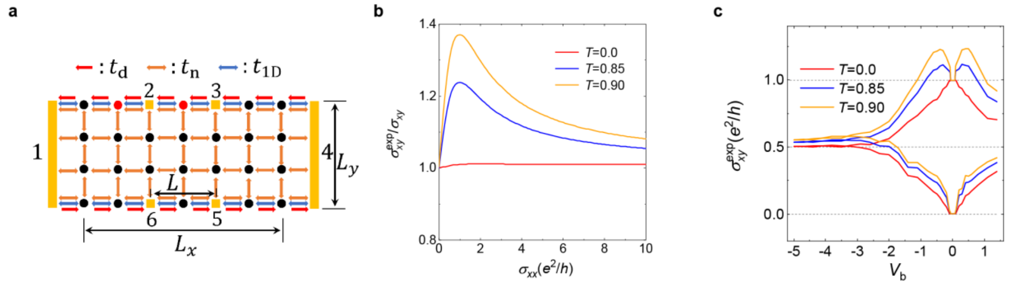

Supplementary Fig. 15 | Numerical calculations of the anomalous Hall conductance affected by quasi-1D helical edge conduction. a, Schematic plot of the network model with sized $L_x \times L_y$=600× 100. **b,** $\sigma_{xy}^{\exp}/\sigma_{xy}$ as function of t_n with helical mode number $M = 4$ and $t_c = 1$. **c,** $\sigma_{xy}^{\exp}$ as function of V_b for different transmission probability T of quasi-1D helical edge channels.

Most of the transport data discussed in the study originate from the topological surface states on the top, bottom, and side surfaces. In order to reveal the role of bulk carriers, we deliberately decrease the Bi:Sb ratio η of the CBV devices (m = 10 QL, x = 0.19, y = 0.11) so that they shift to the hole-doped regime. Due to the reduced thickness, the hole-doping introduced by Cr is also heavier. The overall transport behaviors of the

more heavily hole-doped devices are very similar to that of normal devices, except that they do not exhibit well quantized AHC plateaus.

As shown in Supplementary Fig. 16, two CBV devices with η = 0.73 and 0.62 does behave like a QAH with $\sigma_{xy} = \pm e^2/h$ plateaus near V_{bg} = 100 V. However, they are much less robust comparing to other devices. Due to the stronger coupling between top and bottom magnetic layers (reduced m = 10 and RKKY mechanism from more carriers in the bulk), the 2-step feature as shown in Fig.2a in the main text now degrades into a loop with 2 kinks near $\sigma_{xy} = 0$. When V_{bg} is tuned towards -200 V, the loops with kinks evolve into single loops, indicating the vanished Hall contribution from the bottom surface states. However, because of the bulk carriers, the AHC now becomes much smaller than $e^2/2h$. Another possible explanation is that the Fermi level at the top VBST layers is near the bottom edge of the exchange gap, such that the bottom gate voltage can drive the Fermi level to slightly crossing the valence bands, giving rise to the reduced AHC.

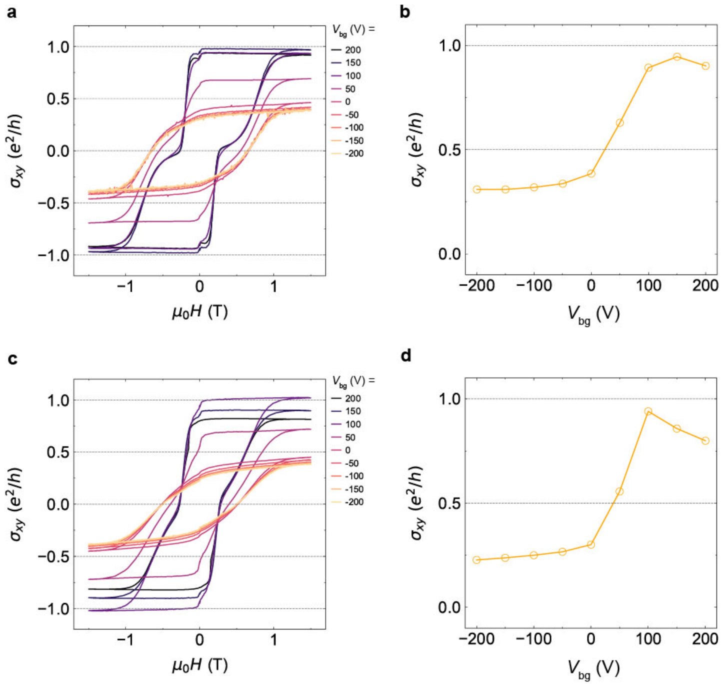

Supplementary Fig. 16 | Deviation from HQLHE in the presence of bulk carriers. **a,** Dependence of the Hall conductance σ_{xy} on the magnetic field $\mu_0 H$ of Device A11

(m = 10 QL, x = 0.19, y = 0.11, η = 0.73), measured at different bottom gate voltage V_{bg}. **b,** The dependence of the zero field Hall conductance on V_{bg} with parallel magnetization configuration of Device A11. **c,** Dependence of the Hall conductance σ_{xy} on the magnetic field $\mu_0 H$ of Device A12 (m = 10 QL, x = 0.19, y = 0.11, η = 0.62), measured at different bottom gate voltage V_{bg}. **d,** The dependence of the zero field Hall conductance on V_{bg} with parallel magnetization configuration of Device A12.

xiii. Discussion on other AHC mechanisms

Contributions to the full AHC can be characterized into three main kinds: intrinsic, skew scattering, and side-jump.[9] As described in the main text, the HQLHE exhibits significant quantized AHC, which can be naturally explained by the giant Berry curvatures of the topological surface bands (intrinsic AHC $\sigma_{xy}^{\mathrm{int}}$).

The skew scattering contribution $\sigma_{xy}^{\mathrm{skew}}$ starts to play a role when the sample is in the "clean limit", usually with large mean-free path and high-density carriers[10]. The devices in this study with magnetic doping on top and bottom surfaces and near the CNP does not satisfy the "clean limit" condition ($\tau \gg \hbar/\varepsilon_F$, τ is the relaxation time, ε_F is the Fermi energy defined with respect to the CNP). The longitudinal conductance σ_{xx} at $V_{\mathrm{bg}} = -200\ \mathrm{V}$ is around $3.8\, e^2/h$ for a film with ~26 nm thickness, which is much smaller comparing to typical skew scattering dominated case $\sigma_{xx} > 10^6 (\Omega cm)^{-1}$. Moreover, $\sigma_{xy}^{\mathrm{skew}}$ is proportional to σ_{xx} ,which clearly does not apply in our cases where the AHC near half-quantization is independent of σ_{xx}.

The side jump contribution $\sigma_{xy}^{\mathrm{sj}}$ is rather complicated in the sense that it is also independent of σ_{xx} and is closely related to the intrinsic Berry curvature in materials with strong spin-orbit coupling. The strong consistency between the experimental AHE data and theoretical models serves as compelling evidence that the intrinsic Berry curvature is the dominant contributor to the observed AHE signals.

We conclude that the dominating mechanism of AHC in this study is intrinsic, similar to the metallic diluted magnetic semiconductor systems, as confirmed by experiments[11]. However, apart from measurement errors, the AHC near the HQLHE regime will be half-quantized only when all the AHC contributions from the bottom surface vanish, which is not always the case in reality. Some of the CBV devices have some extra features near the coercive field of Cr-doped bottom surface in addition to the HQLHE $\sigma_{xy} = \pm e^2/2h$ plateaus. Supplementary Fig. 17 shows several examples of magnetic field scan of Hall and longitudinal traces with the minor loops of Cr-doped bottom surfaces (locate near $\sigma_{xy} = \pm e^2/2h$) having opposite anomalous Hall sign comparing to the major loops.

We attribute this effect to the AHC sign reversal effect in magnetic topological insulators, which stems from the intrinsic Berry curvature. First principle calculations of the hole-doped regime of magnetic TI reveal that the band structures and corresponding AHC can be quite complex[12]. This sign reversal effect can be generated by introducing an electric field, which is consistent with our observation that it only shows up at large negative bottom gate voltage (strongly hole doped). It also seems challenging to precisely predict at what conditions this effect would occur. Here we show 3 examples in Supplementary Fig. 17. The size of this reversed AHC is expected to be affected by multiple factors, including the actual valence band structure, electric field by the bottom gate voltage, built-in electric field at the interfaces, etc. Nevertheless, comparing to the large AHC in the HQLHE, this AHC sign reversal effect is quite small (as in Supplementary Fig. 17b and e) and often negligible (as in Supplementary Fig. 17h and all other devices presented in this study).

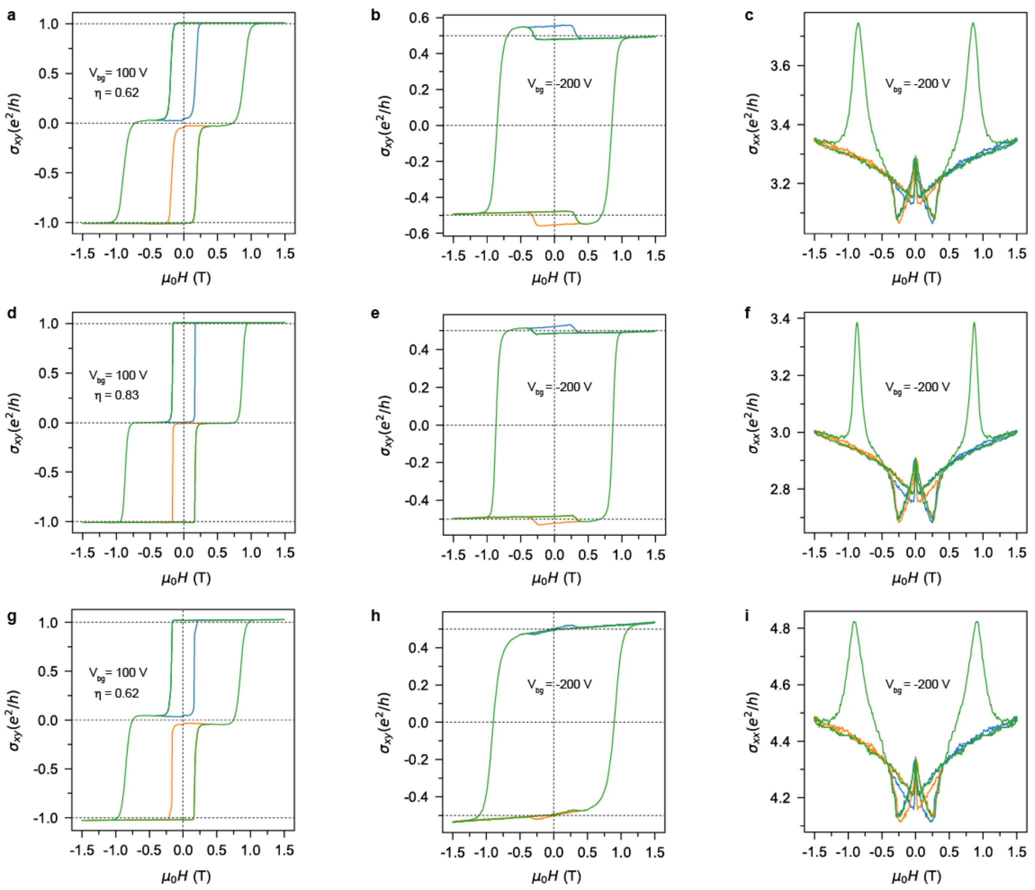

Supplementary Fig. 17 | Small contributions from AHC sign reversal effect. Device A13 (m = 20 QL, x = 0.19, y = 0.11, η = 0.62): **a, b,** Major and minor loops of Hall conductance σ_{xy} vs magnetic field at (**a**) $V_{\mathrm{bg}} = 100$ V, (**b**) $V_{\mathrm{bg}} = -200$ V. **c,** Longitudinal conductance σ_{xx} vs magnetic field at $V_{\mathrm{bg}} = -200$ V. Device A14 (m = 20 QL, x = 0.19, y = 0.11, η = 0.83): **d, e,** Major and minor loops of Hall conductance σ_{xy} vs magnetic field at (**d**) $V_{\mathrm{bg}} = 100$ V, (**e**) $V_{\mathrm{bg}} = -200$ V. **f,** Longitudinal conductance σ_{xx} vs magnetic field at $V_{\mathrm{bg}} = -200$ V. Device A15 (m = 20 QL, x = 0.19, y = 0.11, η = 0.62): **g, h,** Major and minor loops of Hall conductance σ_{xy} vs magnetic field at (**g**) $V_{\mathrm{bg}} = 100$ V, (**h**) $V_{\mathrm{bg}} = -200$ V. **i,** Longitudinal conductance σ_{xx} vs magnetic field at $V_{\mathrm{bg}} = -200$ V.

xiv. Comparative analysis vs. layer Hall effect and the parity anomaly state

While it is well established that a single massive Dirac surface yields $\sigma_{xy} = e^2/2h$[14], and that layer-polarized transport has been proposed[15], our result cannot be reduced to a trivial combination of these ideas. The realization of half-quantized layer Hall effect (HQLHE) within a fully tunable, compensated magnetic system, provides the first direct boundary probe of the bulk axion field.

Until now, no explicit theoretical prediction of the HQLHE existed. In fact, a very recent calculation on similar systems[16] predicted a maximum layer Hall conductance significantly smaller than the half-quantized value. This discrepancy highlights a critical gap in theoretical understanding. This study, through experiment and modeling, help resolve this by identifying new physical mechanisms—disorder-mediated stabilization and bulk trap state screening—which are essential for achieving robust quantization. This finding transforms this phenomenon from a trivial combination of LHE[15] and parity anomaly state[17] into an unexpected discovery.

Below we compare this HQLHE result with these two related prior studies and summarize the main differences in Supplementary Table 1:

vs. Gao *et al.*[15]: First, we demonstrate the first quantized layer Hall effect, a monumental advance from their non-quantized result. The transition from a non-quantized to a quantized Hall response encoded in the layer degree of freedom is comparable in significance to the historical leap from the classical Hall effects (charge or spin) to their quantum counterparts. Second, our material-by-design approach (CBST/BST/VBST) naturally breaks $\mathcal{PT}$-symmetry through tailored doping profiles, enabling quantization without an external electric field in some devices (e.g., Fig. 2a). The underlying physical mechanism is very different: driven by external electric field, or by Fermi level positioning (this work). Third, this experimental realization required overcoming substantial challenges, such as identifying a suitable spacer thickness (10–20 QLs) to suppress both surface hybridization and excessive conduction channels, achieving precise control of dual magnetic exchange gaps, and establishing asymmetric Fermi level positioning. These complexities, while underscoring the tunability and versatility of this material platform, also highlight that the complete extraction of a half-quantized Hall signal from a single surface is physically non-trivial.

vs. Mogi *et al.*[17]: The system and scientific goals of this study are fundamentally different. The fully magnetic, compensated antiferromagnetic system realized in this study can be utilized to probe the quantized axion field through layer Hall effect, while the semi-magnetic system, lack of layer degree of freedom, cannot host axion insulator phase. Notably, signatures of deviation from half-quantization in Mogi *et al.*[17], such as AHC peak near the CNP and smaller values in more strongly doped regimes (e.g. Supplementary Fig. 10C in their supplementary material), are absent in this work. The HQLHE occurs only in strongly doped regime and sometimes even exceeds $e^2/2h$ slightly. These discrepancies strongly suggest that the two half-quantization effects arise from distinct mechanisms. Our theoretical framework, as discussed in Supplementary Note xii, demonstrates that half-quantization emerges when a classical metallic state forms on the conducting surface, a condition fulfilled in our system through magnetic disorder and strong doping, but not necessarily in the undoped, gapless Dirac state studied by Mogi *et al*. In addition, the microscopic origin of the half-quantization in semi-magnetic systems is still under active theoretical debate, with some theoretical studies[18] suggesting that the parity anomaly state in the semi-magnetic system stems from the gapless surface rather than the gapped one.

Supplementary Table 1 | Comparative Analysis of previous Works and the Present Work

**Feature**	**Gao *et al.* (*Nature*, 2021)**	**Mogi *et al.* (*Nat. Phys.*, 2022)**	**Present Work**
**System**	Even-layered $MnBi_2Te_4$ (antiferromagnetic axion insulator)	Semi-magnetic TI heterostructure with one magnetically doped surface	Magnetically doped TI heterostructure (prototypical axion insulator)
**Symmetry**	$\mathcal{PT}$-symmetric ground state	Broken inversion and time-reversal symmetry by design	Antiferromagnetic ground state (axion insulator)
**Control**	Perpendicular electric	Static material	Asymmetric

**Mechanism**	field to break $\mathcal{PT}$-symmetry	engineering; Fermi level tuned to pre-existing gap	electrostatic gating to tune Fermi level of individual layers
**Nature of the anomalous Hall effect**	Layer-polarized AHC, **non-quantized**	Half-quantized AHC ($\sigma_{xy} = e^2/2h$)	Layer-resolved, **precisely quantized** AHC ($\sigma_{xy} = e^2/2h$)
**Conceptual Advance**	First observation of a layer-polarized Hall effect	Realization of parity anomaly in a single massive Dirac fermion system	First realization of a **quantized Layer Hall Effect,** a boundary signature of the quantized axion field

xv. Fermi level tuning efficiency by the bottom gate voltage

To elucidate the role of back-gate voltage V_{bg}, we derive the relationship between the chemical potential $\mu(\mathrm{z})$ and V_{bg}. We find that the chemical potential of top (μ_{t}) and bottom (μ_{b}) surface satisfy $\mu_{\mathrm{t}}(V_{\mathrm{bg}}) = \mu_{\mathrm{b}}(V_{\mathrm{bg}})/\cosh(d/\lambda)$, where d is the thickness of the magnetic TI and $\lambda = \sqrt{\varepsilon/(e^2 D_0)}$ is the Thomas–Fermi screening length. Here, D_0 denotes the density of bulk trap states and ε is the absolute permittivity of TI. In the limit $d \gg \lambda$, the top-surface chemical potential vanishes ($\mu_{\mathrm{t}}\,(V_{\mathrm{bg}}) \approx 0$). The high density of bulk trap states in the magnetic TI effectively screens electric fields in the bulk, thereby the back gate only tunes the chemical potential of the bottom surface. Consistently, the electrostatic potential takes the form $\phi(z) = \phi_{\mathrm{b}}\cosh((d-z)/\lambda)/\cosh(d/\lambda)$. $\phi(\mathrm{z})$ in the bulk also approaches zero as $d \gg \lambda$, and the back-gate voltage only changes the potential of the bottom surface (ϕ_{b}). When $e|\phi_{\mathrm{b}}| <$ bulk band gap Δ_{bulk} of magnetic TI, the back-gate voltage V_{bg} mainly tunes the chemical potential of bottom surface. When $e|\phi_{\mathrm{b}}| \sim\Delta_{\mathrm{bulk}}$, however, V_{bg} starts to modify the

bottom-surface band structure. In our experiments, the bulk gap is $\Delta_{\mathrm{bulk}} \approx 300$ meV[19], while the surface magnetization gap is only $M_{\mathrm{b}} \approx 1$ meV. Therefore, when the system realizes the half-quantized layer Hall effect (e.g., $|\mu_{\mathrm{b}}| = e|\phi_{\mathrm{b}}| \gg M_{\mathrm{b}}$), the condition $e|\phi_{\mathrm{b}}| \ll \Delta_{\mathrm{bulk}}$ is still satisfied, and V_{bg} predominantly controls the bottom-surface chemical potential in this regime. Note that even if the bottom-surface band structure is slightly modified, it contributes negligible Berry curvature once the chemical potential is tuned deep into the metallic regime.

Therefore, for thick magnetic TIs with a high density of bulk trap states, the back-gate voltage V_{bg} serves primarily to locally tune the electrostatic and chemical potential of the bottom surface. In practice, this allows the back-gate to shift the chemical potential of the bottom surface sufficiently far into a metallic regime, while the top surface, subject to a much weaker effective gate field, remains within its local magnetic gap. In current study, the exact density of bulk trap states is unknown. By assuming that the conductance deviation is mainly caused by the insufficient electrostatic screening, we roughly estimate from the data of Device A4 (spacer layer thickness $m = 5$) that Thomas–Fermi screening length in our samples is $\lambda \sim 5$ nm. This corresponds to a 3-dimensional density of bulk trap states $D_0 = \varepsilon/(e\lambda)^2 = 5.5 \times 10^{44}\ m^{-3}J^{-1}$, about 15 times larger than the measured $D_0 = 3.6 \times 10^{43}\ m^{-3}J^{-1}$ by capacitance spectroscopy of a 67 nm $BiSbTeSe_2$ thin flake exfoliated from crystals grown by the modified Bridgman method[20]. This is a reasonable value considering the non-equilibrium growth nature and the active magnetic doping by molecular beam epitaxy, which inevitably introduce much more defects related to bulk trap states.

Below we derive the relationship between the chemical potential of the top and bottom surfaces under gating conditions.

We consider a magnetic topological insulator with a back gate, which extends infinitely in the *x* and *y* directions. The electrochemical potential is defined as $\mu^{\mathrm{e}}(z) = -e\phi(z) + \mu(z)$ where $\phi(\mathrm{z})$ is the electrostatic potential and $\mu(\mathrm{z})$ is the chemical potential. When the TI is grounded (i.e. $\mu^{\mathrm{e}}(z) = 0$), it follows that $\mu(\mathrm{z}) = e\phi(\mathrm{z})$. In addition, the magnetic TIs in our experiments host bulk trap states. For simplicity, we

take the density of states of these bulk trap states, D_0, to be constant. In the bulk, the Poisson equation is

$$\varepsilon \partial_z^2 \phi(z) = eD_0\mu(z) = e^2 D_0 \phi(z), \tag{12}$$

where ε is the absolute permittivity of the magnetic TI. The solution is given by $\phi(z) = A_1 e^{-z/\lambda} + A_2 e^{z/\lambda}$, where $\lambda = \sqrt{\varepsilon/(e^2 D_0)}$ is the Thomas–Fermi screening length. We next consider the boundary conditions of the electric field at the upper and lower interfaces of the TI. They are given by

$$\begin{cases} \varepsilon E_z(0) = C_{\mathrm{bg}}\left(\frac{\mu_{\mathrm{b}}}{e} - V_{\mathrm{bg}}\right) - en_{\mathrm{b}}(\mu_{\mathrm{b}}) \\ -\varepsilon E_z(d) = -en_{\mathrm{t}}(\mu_{\mathrm{t}}) \end{cases} \tag{13}$$

Here, $\mu_{\mathrm{b}} = \mu(0) = eA_1 + eA_2$ and $\mu_{\mathrm{t}} = \mu(d) = eA_1 e^{-d/\lambda} + eA_2 e^{d/\lambda}$ are the chemical potential of the top and bottom surface, respectively. Here, we set $z = 0$ as the bottom surface, and $z = d$ as the top surface of the TI. $n_i(\mu) = \frac{(\mu^2 - M_i^2)}{4\pi(\hbar v_F)^2}\,\mathrm{sgn}(\mu)\,\Theta(|\mu| - |M_i|)$ with $i = \mathrm{t}, \mathrm{b}$, is the electron density of the surface states, and v_F is the Fermi velocity. Substituting electric field $E_z(z) = -\partial_z \phi(z)$ into these equations, we obtain

$$\begin{cases} C_{\mathrm{bulk}}(-A_1 + A_2) = C_{\mathrm{bg}}\left(\frac{\mu_{\mathrm{b}}}{e} - V_{\mathrm{bg}}\right) - en_{\mathrm{b}}(\mu_{\mathrm{b}}) \\ -C_{\mathrm{bulk}}\left(-A_1 e^{-d/\lambda} + A_2 e^{d/\lambda}\right) = -en_{\mathrm{t}}(\mu_{\mathrm{t}}) \end{cases} \tag{14}$$

where $C_{\mathrm{bulk}} = \varepsilon/\lambda$ is the bulk capacitance of the trap states while C_{bg} is the capacitance of the back gate. By solving these equations, we can get A_1 and A_2 as function of back gate voltage V_{bg}. This, in turn, allows us to get μ_{t} and μ_{b}.

When $|\mu_{\mathrm{t}}| < M_{\mathrm{t}}, n_{\mathrm{t}}(\mu_{\mathrm{t}}) = 0$ and $A_2 = A_1 e^{-2d/\lambda}$, then we have

$$\mu_{\mathrm{t}} = \mu_{\mathrm{b}}/\cosh(d/\lambda), \tag{15}$$

while the electrostatic potential takes the form $\phi(\mathrm{z}) = \phi_{\mathrm{b}}\cosh((d - z)/\lambda)/\cosh(d/\lambda)$ with the bottom surface electrostatic potential ϕ_b. When the magnetic TI is sufficiently thick or the density of bulk trap states is large, i.e., $d \gg \lambda$, the top-surface chemical potential satisfies $\mu_t \approx 0$. In this regime, the back gate effectively tunes only the chemical potential of the bottom surface. The high density of bulk trap states in the magnetic TI strongly screens the electric field in the bulk, thereby pinning the top-

surface chemical potential within the magnetization gap.

xvi. Additional raw data of the CBV devices

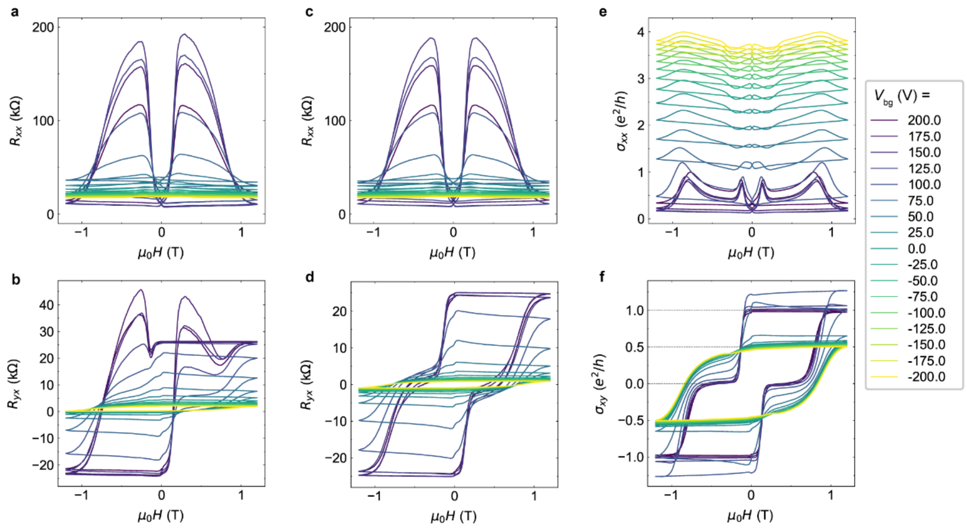

Supplementary Fig. 18 | Symmetrization and anti-symmetrization of the magnetic field scan data for Device A4. a, b, The original R_{xx} and R_{yx} vs magnetic field data, measured at various bottom gate voltage V_{bg}. **c, d,** R_{xx} and R_{yx} vs magnetic field, after symmetrization and anti-symmetrization, respectively. **e, f,** σ_{xx} and σ_{xy} vs magnetic field calculated from processed data in **c** and **d**.

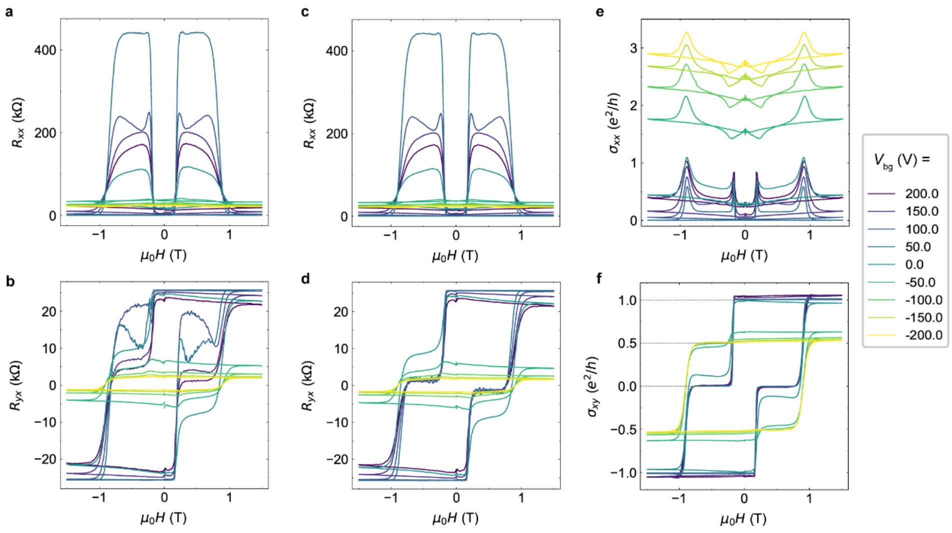

Supplementary Fig. 19 | Symmetrization and anti-symmetrization of the magnetic field scan data for Device A5. a, b, The original R_{xx} and R_{yx} vs magnetic field data, measured at various bottom gate voltage V_{bg}. **c, d,** R_{xx} and R_{yx} vs magnetic field, after symmetrization and anti-symmetrization, respectively. **e, f,** σ_{xx} and σ_{xy} vs magnetic field calculated from processed data in **c** and **d**.

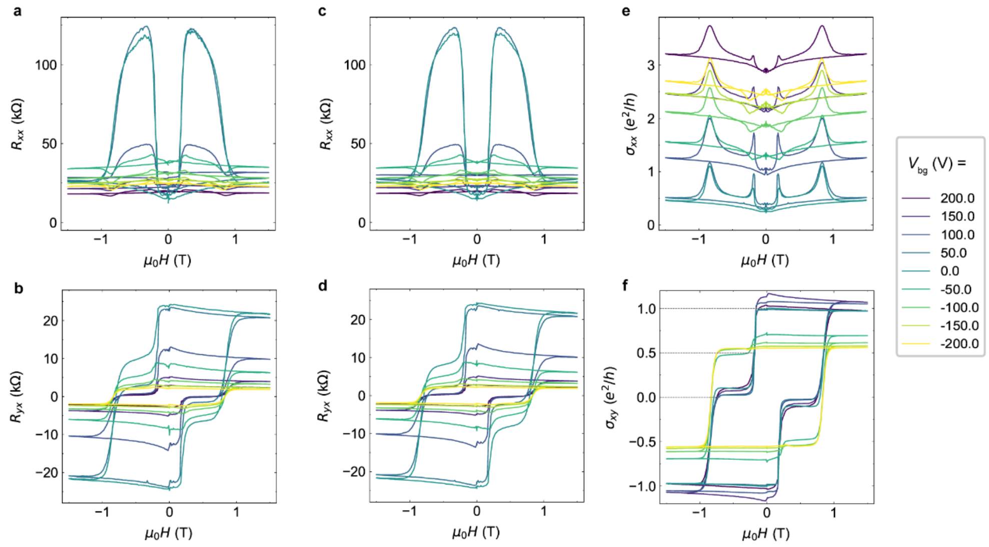

Supplementary Fig. 20 | Symmetrization and anti-symmetrization of the magnetic field scan data for Device A6. a, b, The original R_{xx} and R_{yx} vs magnetic field data, measured at various bottom gate voltage V_{bg}. **c, d,** R_{xx} and R_{yx} vs magnetic field, after symmetrization and anti-symmetrization, respectively. **e, f,** σ_{xx} and σ_{xy} vs magnetic field calculated from processed data in **c** and **d**.

xvii. Characteristics of all devices measured in this study

All measurements of the AHC σ_{xy} were performed at $T = 30\,\mathrm{mK}$ and $V_{\mathrm{bg}} = -200\,\mathrm{V}$ except for Device C1, E1 and E2 ($V_{\mathrm{bg}} = 200\,\mathrm{V}$). The averaged AHC of HQLHE in the main text with $\sigma_{xy} = 0.505 \pm 0.019\ e^2/h$ is calculated from the data in bold (devices with spacer thickness $10 \leq m \leq 20$ QLs). Detailed characteristics of all devices in this study are summarized in the table below.

Supplementary Table 2 | Characteristics of all devices measured in the study.

**Devices in the text**	**Device structures**	m	x	y	η	σ_{xy} (e^2/h) **(parallel)**	σ_{xy} (e^2/h) **(antiparallel)**
Device A1 (Fig. 1 & 2)	CBV	20	0.19	0.11	0.83	**0.507**	**0.521**
Device B1 (Fig. 3)	CBV (dual-gated)	20	0.19	0.11	0.83	0.463	0.446
Device C1 (Fig. 4)	VBC	20	0.19	0.03	0.73	0.522	/
Device A2 (Fig. 4 & Supplementary Fig.5)	CBV	20	0.19	0.11	0.83	**0.471**	**0.504**
Device A3 (Supplementary Fig. 1)	CBV	20	0.36	0.11	0.83	0.768	0.351
Device A4 (Supplementary Fig. 3 & 18)	CBV	5	0.19	0.11	0.83	0.455	0.479
Device A5 (Supplementary Fig. 3, 9 & 19)	CBV	10	0.19	0.11	0.83	**0.511**	**0.522**
Device A6 (Supplementary Fig. 3 & 20)	CBV	30	0.19	0.11	0.83	0.554	0.565
Device A7 (Supplementary Fig. 5)	CBV	20	0.19	0.11	0.83	**0.497**	**0.501**
Device A8 (Supplementary Fig. 5)	CBV	20	0.19	0.11	0.62	**0.486**	**0.519**
Device D1 (Supplementary Fig. 8)	CBC	10	0.19	0.11	0.83	0.515	/

Device D2 (Supplementary Fig. 8)	CBC	20	0.19	0.11	0.83	0.538	/
Device A9 (Supplementary Fig. 9)	CBV	20	0.19	0.23	0.83	0.533	0.557
Device A10 (Supplementary Fig. 14)	CBV	20	0.19	0.11	0.73	0.764	/
Device A11 (Supplementary Fig. 16)	CBV	10	0.19	0.11	0.73	0.301	/
Device A12 (Supplementary Fig. 16)	CBV	10	0.19	0.11	0.62	0.227	/
Device A13 (Supplementary Fig. 17)	CBV	20	0.19	0.11	0.62	**0.478**	**0.552**
Device A14 (Supplementary Fig. 17)	CBV	20	0.19	0.11	0.83	**0.496**	**0.519**
Device A15 (Supplementary Fig. 17)	CBV	20	0.19	0.11	0.62	**0.494**	**0.498**
Device E1 (Supplementary Fig. 6)	CBV	20	0.19	0.11	see related caption	0.744	/
Device E2 (Supplementary Fig. 6)	CBV	20	0.19	0.11		0.529	/

Supplementary References

1. Akkermans, E. & Montambaux, G. *Mesoscopic Physics of Electrons and Photons*. (Cambridge University Press, 2007).
2. Kirkpatrick, S. Classical Transport in Disordered Media: Scaling and Effective-Medium Theories. *Phys. Rev. Lett.* **27**, 1722–1725 (1971).
3. Erdös, P. & Haley, S. B. Random-network models of the conductance of disordered condensed matter. *Phys. Rev. B* **13**, 1720–1727 (1976).

4. Zhou, H., Chen, C.-Z., Sun, Q.-F. & Xie, X. C. Dissipative chiral channels, Ohmic scaling, and half-integer Hall conductivity from relativistic quantum Hall effect. *Phys. Rev. B* **109**, 115305 (2024).
5. Zhou, H. *et al.* Transport Theory of Half-Quantized Hall Conductance in a Semimagnetic Topological Insulator. *Phys. Rev. Lett.* **129**, 096601 (2022).
6. Buttiker, M. Symmetry of electrical conduction. *IBM Journal of Research and Development* **32**, 317–334 (1988).
7. Liu, Y., Bian, G., Miller, T., Bissen, M. & Chiang, T.-C. Topological limit of ultrathin quasi-free-standing Bi_2Te_3 films grown on Si(111). *Phys. Rev. B* **85**, (2012).
8. Zhuo, D. *et al.* Axion insulator state in hundred-nanometer-thick magnetic topological insulator sandwich heterostructures. *Nat. Commun.* **14**, 7596 (2023).
9. Nagaosa, N., Sinova, J., Onoda, S., MacDonald, A. H. & Ong, N. P. Anomalous Hall effect. *Rev. Mod. Phys.* **82**, 1539–1592 (2010).
10. Onoda, S., Sugimoto, N. & Nagaosa, N. Intrinsic Versus Extrinsic Anomalous Hall Effect in Ferromagnets. *Phys. Rev. Lett.* **97**, 126602 (2006).
11. Chun, S. H. *et al.* Interplay between Carrier and Impurity Concentrations in Annealed $Ga_{1-x}Mn_xAs$: Intrinsic Anomalous Hall Effect. *Phys. Rev. Lett.* **98**, 026601 (2007).
12. Wang, F. *et al.* Interface-induced sign reversal of the anomalous Hall effect in magnetic topological insulator heterostructures. *Nat. Commun.* **12**, 79 (2021).
13. Jain, R. *et al.* A quantized anomalous Hall effect above 4.2 K in stacked topological insulator/magnet bilayers. Preprint at http://arxiv.org/abs/2412.05380 (2024).
14. Niemi, A. J. & Semenoff, G. W. Axial-Anomaly-Induced Fermion Fractionization and Effective Gauge-Theory Actions in Odd-Dimensional Space-Times. *Phys. Rev. Lett.* **51**, 2077–2080 (1983).
15. Gao, A. *et al.* Layer Hall effect in a 2D topological axion antiferromagnet. *Nature* **595**, 521–525 (2021).
16. Han, Y., Guo, Y., Li, Z. & Qiao, Z. Layer Hall Effect without External Electric Field. *Phys. Rev. Lett.* **134**, 236206 (2025).
17. Mogi, M. *et al.* Experimental signature of the parity anomaly in a semi-magnetic topological insulator. *Nat. Phys.* **18**, 390–394 (2022).
18. Zou, J.-Y. *et al.* Half-quantized Hall effect at the parity-invariant Fermi surface. *Phys. Rev. B* **107**, 125153 (2023).
19. Zhang, H. *et al.* Topological insulators in Bi_2Se_3, Bi_2Te_3 and Sb_2Te_3 with a single Dirac cone on the surface. *Nat. Phys.* **5**, 438–442 (2009).
20. Wang, J. *et al.* Two-Dimensional-Dirac Surface States and Bulk Gap Probed via Quantum Capacitance in a Three-Dimensional Topological Insulator. *Nano Lett.* **20**, 8493-8499 (2020).
